# The value of baseline 18F-sodium fluoride and 18F-choline PET activity for identifying responders to radium-223 treatment in castration-resistant prostate cancer bone metastases

**DOI:** 10.1007/s00330-023-10172-7

**Published:** 2023-08-24

**Authors:** Ricardo Donners, Nina Tunariu, Holly Tovey, Emma Hall, Sue Chua, Gary Cook, Yong Du, Matthew D. Blackledge, Christopher C. Parker, Dow-Mu Koh

**Affiliations:** 1 Department of Radiology, University Hospital Basel, Petersgraben 4, 4031 Basel, Switzerland; 2 The Institute of Cancer Research, 15 Cotswold Road, Sutton, SM2 5NG UK; 3 Royal Marsden Hospital, Downs Road, Sutton, SM2 5PT UK; 4 King’s College London and Guy’s and St. Thomas’ PET Centre, St. Thomas’ Hospital, King’s College London, Westminster Bridge Rd, London, UK

**Keywords:** Metastases, Radium, Bone, Positron emission tomography computed tomography, Diffusion magnetic resonance imaging

## Abstract

**Objectives:**

To investigate whether baseline 18F-sodium fluoride (NaF) and 18F-choline PET activity is associated with metastatic castration-resistant prostate cancer (mCRPC) global and individual bone metastases’ DWI MR imaging response to radium-223 treatment.

**Methods:**

Thirty-six bone-only mCRPC patients were prospectively recruited from three centers. Whole-body (WB)-MRI with DWI and 18F-NaF and 18F-choline PET/CT were performed at therapy baseline and 8-week intervals. In each patient, bone disease median global (g)ADC change between baseline and follow-up was calculated. Additionally, up to five bone target lesions per patient were delineated and individual median ADC change recorded. An ADC increase > 30% defined response per-patient and per-lesion. For the same targets, baseline 18F-NaF and 18F-choline PET SUVmax were recorded. Mean SUVmax across patient targets was correlated with gADC change and lesion SUVmax with per-lesion ADC change.

**Results:**

A total of 133 lesions in 36 patients (14 responders) were analyzed. 18F-NaF PET per-patient mean SUVmax was significantly higher in responders (median = 56.0 versus 38.7 in non-responders; *p* = 0.008), with positive correlation between SUVmax and gADC increase (rho = 0.42; *p* = 0.015). A 48.7 SUVmax threshold identified responders with 77% sensitivity and 75% specificity. Baseline 18F-NaF PET per-lesion SUVmax was higher in responding metastases (median = 51.6 versus 31.8 in non-responding metastases; *p* = 0.001), with positive correlation between baseline lesion SUVmax and ADC increase (rho = 0.39; *p* < 0.001). A 36.8 SUVmax threshold yielded 72% sensitivity and 63% specificity. No significant association was found between baseline 18F-choline PET SUVmax and ADC response on a per-patient (*p* = 0.164) or per-lesion basis (*p* = 0.921).

**Conclusion:**

18F-NaF PET baseline SUVmax of target mCRPC bone disease showed significant association with response to radium-223 defined by ADC change.

**Clinical relevance statement:**

18F-sodium fluoride PET/CT baseline maximum SUV of castration-resistant prostate cancer bone metastases could be used as a predictive biomarker for response to radium-223 therapy.

**Key Points:**

*• 18F-sodium fluoride PET baseline SUVmax of castration-resistant prostate cancer bone metastases showed significant association with response to radium-223.*

*• Baseline 18F-sodium fluoride PET can improve patient selection for radium-223 therapy.*

*• Change in whole-body DWI parameters can be used for response correlation with baseline 18F-sodium fluoride PET SUVmax in castration-resistant prostate cancer bone metastases.*

## Introduction

Metastatic castrate-resistant prostate cancer (mCRPC) is a fatal disease with a mean overall survival between 18 and 36 months [1]. Ninety percent of mCRPC patients develop bone metastases, and in up to 45% the skeleton is the only site of spread [2]. Bone metastases can cause fractures and cord compression, which are major contributors to morbidity and mortality [3]. Although recent therapeutic developments have significantly increased patient survival, treatment options remain limited [4]. A promising therapeutic agent is the bone-seeking alpha emitter radium-223, which can prolong patient survival and delay skeletal events [4, 5]. However, patients’ benefit and outcome are strongly influenced by patient selection [4, 6, 7]. Consequently, baseline imaging parameters in mCRPC bone metastases, which may predict response to radium-223 therapy, are desirable. 

WB-MRI with diffusion-weighted imaging (WB-DWI) and 18F-choline and 18F-sodium fluoride (NaF)-PET/CT outperform conventional CT, MRI, and bone scintigraphy for disease detection and staging in mCRPC patients with bone disease [8–11]. DWI and PET/CT allow for quantitative lesion measurements beyond tumor size, which may serve as imaging biomarkers. The most common parameters measured are the DWI apparent diffusion coefficient (ADC), which quantifies tissue water mobility and inversely correlates with tumor tissue cellularity [12], and the PET standardized uptake value (SUV), quantifying radioactive tracer tissue activity. The ADC and maximum SUV (SUVmax) have good measurement repeatability [13–15].

WB-DWI guidelines, incorporating ADC interpretation, were established for monitoring mCRPC bone disease [14]. Based on contemporary data, an ADC increase ≥ 30% is consistent with a real treatment benefit of bone metastases [14, 15]. While WB-DWI allows for identification of therapy response, 18F-NaF and choline PET/CT may allow for response prediction from baseline imaging. Given the similar uptake properties of radium-223 and 18F-NaF PET tracer in osteoblastic bone [5], a reasonable hypothesis is that metastatic disease with higher baseline SUVmax is more likely to respond to radium-223 therapy, resulting in ADC change > 30%, while less tracer-avid metastases may not. In contrast, increased choline levels can reflect on more aggressive tumor, which may result in poorer treatment response represented by interval ADC change < 30% [16]. Previous studies provided some support for these hypotheses in mCRPC bone metastases [17–22], but direct correlation of baseline PET/CT SUV with WB-DWI ADC interval change during therapy as a surrogate of treatment response has not been reported. 

In this dedicated imaging study, we evaluated whether baseline 18F-NaF and 18F-choline PET SUVmax of mCRPC bone metastases are associated with response to radium-223 on a per-patient and per-lesion basis, defined by the increase in global ADC and lesion ADC.

## Materials and methods

This study is an exploratory imaging analysis conducted as part of a prospective, three-center randomized controlled trial, evaluating the response of chemotherapy-naïve, bone-only mCRPC patients to radium-223. The primary objective of the parent trial was identifying potential imaging response biomarkers. The trial was approved by the research and ethics committee and all patients provided written informed consent. All trial patients were available for inclusion in this dedicated imaging study, which is presented in this manuscript.

### Study population

Thirty-nine men with a median age of 74.5 (IQR 72.1–79.5) years were prospectively recruited from three different oncology clinics between 27.05.2015 and 15.06.2017, and randomly assigned to receive either 88 Bq/kg or 55 Bq/kg of radium-223. The obligatory trial inclusion criteria for these 39 men were as follows: histologically confirmed mCRPC, multiple (> 2) skeletal metastases identified on bone scintigraphy, age > 18 years, life expectancy > 6 months, no prior chemotherapy for CRPC, provision and comprehension of the full trial requirements, and signed informed consent. Exclusion criteria were the following: any prior radioisotope therapy, any anti-cancer therapy within 4 weeks prior to study randomization with exception of luteinizing hormone–releasing hormone agonists, other malignancies diagnosed within 3 years prior to trial randomization, treatment with any investigational drug 30 days prior to randomization, presence or history of visceral mCRPC metastases, malignant lymphadenopathy, known brain or meningeal disease, imminent or established spinal cord compression, blood transfusions, bone marrow stimulating agents within 4 weeks prior to randomization, and general MRI contraindications.

After randomization, up to six cycles of radium-223 were administered intravenously in 4-week intervals.

### Imaging techniques

WB-MRI, 18F-NaF PET/CT, and 18F-choline PET/CT were performed within 14 days prior to the first radium cycle (baseline) and within ± 7 days of the treatment cycles 2 and 4. A minimum of 12 h was allowed between 18F-NaF and 18F-choline PET/CT. End of treatment imaging was performed 4 weeks ± 7 days after the last radium-223 administration. Only the baseline 18F-NaF and 18F-choline PET/CTs were analyzed in the presented study.

MRIs were acquired on 1.5-T Siemens MAGNETOM Aera and MAGNETOM Avanto systems (Siemens Healthineers). WB-MRI was performed from the skull base to mid-thigh comprising DWI (*b*-values of 50 and 900 s/mm^2^) and T1-weighted volume-interpolated breath hold examination (VIBE) Dixon sequences, with matching field of view and slice thickness (Table 1). DWI and VIBE were supplemented by sagittal T1- and T2-weighted turbo spin-echo images of the spine.

**Table 1:** MRI protocol

Parameter	DWI	T1 VIBE Dixon
Plane	Axial	Axial
Slice thickness (mm)	5	5
*b*-values in s/mm^2^	50, 900	-
Field of view (mm)	400 × 390	400 × 390
Acquisition matrix	150 × 144	256 × 105
Repetition time (ms)	14,600	13.9
Echo time (ms)	64.8	2.39
Number of averages	4/*b*-value	1
Flip angle	120°	70°
Bandwidth (Hz/pixel)	1961	470
Acquisition time (min:s)	2:21	0:33

*VIBE* volume-interpolated breath hold examination

PET/CT studies were undertaken on Siemens Biograph systems (Siemens Healthineers). Images were acquired from the vertex to mid-thighs 60 (± 5) min post injection of 250 (± 25) MBq of 18F-NaF or 300 (± 30) MBq of 18F-choline, respectively. A low-dose CT was performed for attenuation correction and image fusion. PET data were reconstructed using an ordered subset expectation maximization algorithm.

### Image analysis

**Fig. 1:**
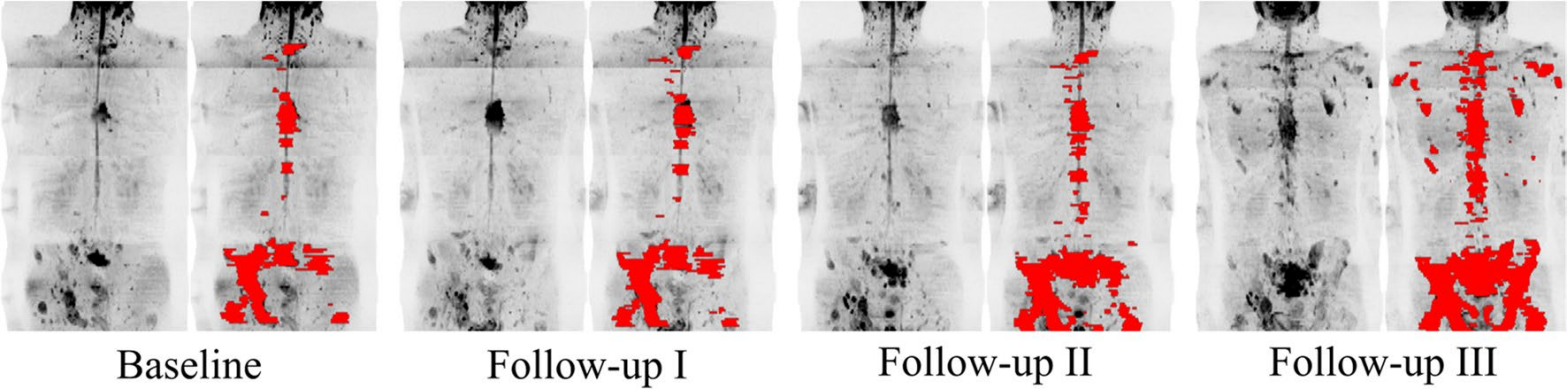
Segmentation of total skeletal disease volume in a 74-year-old metastatic castrate-resistant, non-responding prostate cancer patient on whole-body DWI, maximum intensity projection of the composed *b*900 DWI images with and without superimposed total diffusion volume (red) representing the segmented skeletal disease burden for baseline and three follow-up imaging time-points (I–III)

MRI analysis was conducted on commercially available software (OsiriX, version 56, Pixmeo SARL Bernex) by a board-certified radiologist with 15 years of experience in cancer imaging. The total skeletal disease diffusion volume (tDV) was obtained by manually segmenting all sites of visible high signal intensity bone disease on the *b*900 DWI images. The tDV volumes of interest (VOIs) were transferred onto the corresponding ADC maps to obtain the median global disease ADC (gADC) for each patient at baseline and for each follow-up WB-MRI (Fig. 1). The percentage gADC change between baseline and each follow-up MRI was calculated. Patients with a gADC increase ≥ 30% between baseline and any follow-up MRI were defined as responders. The remaining patients were non-responders. The largest increase between baseline gADC and any follow-up MRI was labeled “best patient response.”

Additionally, up to five target bone metastases, each > 2 cm in axial dimensions, were chosen on WB-DWI baseline imaging, regardless of CT attenuation. These were individually volume-segmented on the *b*900 images using OsiriX. Similar to the soft tissue target lesion selection approach described for RECIST 1.1, larger, representative lesions were chosen, facilitating reliable follow-up measurement [23]. The resulting volume segmentations were copied onto the ADC maps and the median ADC values and lesion diffusion volumes were derived for each metastasis. The process was repeated for the follow-up MRIs and the per-lesion median ADC change was calculated between baseline and each follow-up. Any target lesion showing a median ADC increase ≥ 30% was defined as a responding metastasis, and < 30% as non-responding. The largest increase between target baseline ADC and any follow-up MRI was labeled “best target lesion response.”

**Fig. 2:**
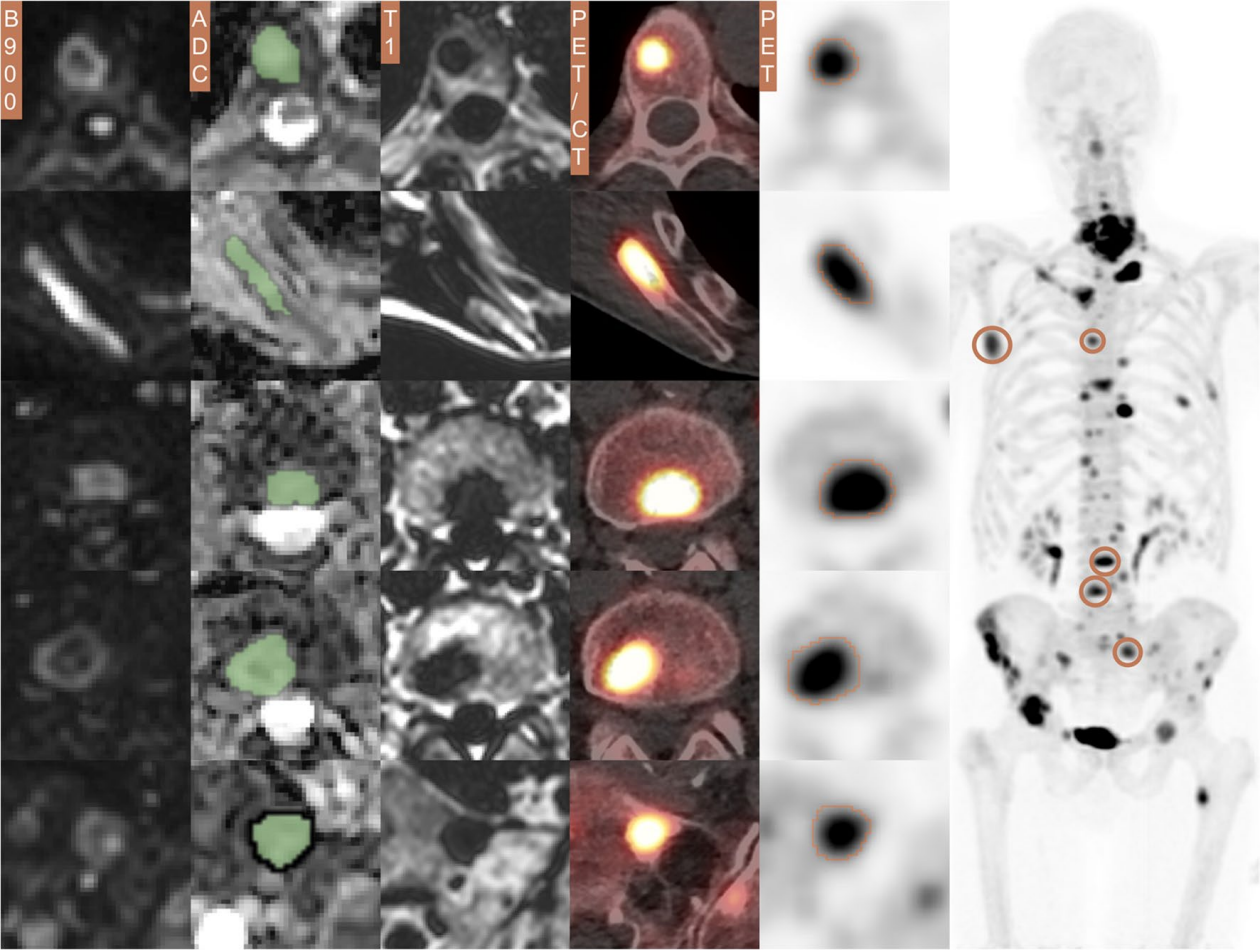
Target lesion measurements in a 65-year-old metastatic castrate-resistant prostate cancer patient, 5 target lesions on *b*900 DWI, ADC (with green segmentation), T1-weighted and 18F-NaF PET/CT fusion, and PET (delineated) images; apparent difference in lesion size between DWI and PET images may relate to the difference in functional properties used to generate image contrast, variance in patient positioning, and slice selection as well as difference in resolution of these two imaging techniques

The same five target lesions identified on WB-MRI were delineated as VOIs on the respective baseline 18F-NaF and 18F-choline PET images using HERMES Gold software (Hermes Medical Solutions, Inc.) (Fig. 2). For each metastasis, the SUVmax was recorded. For per-patient analyses, the average value across all targets was calculated.

### Statistical analyses

Statistical analyses were performed using Stata v16.1. The Shapiro–Wilk test was used to identify normal distribution of SUVmax average and individual target measurements. In case of normal distribution *t*-tests, in the absence of normal distribution, the Mann–Whitney *U* tests were performed to compare baseline SUVmax values between MRI responders and non-responders. In case of significant difference, ROC AUC analysis was performed. The Youden index facilitated choice of optimized SUVmax threshold values to distinguish between responders and non-responders. Additionally, Spearman rank correlation coefficients were calculated between percentage ADC change and baseline SUVmax values.

Analyses were performed both on a per-patient and per-lesion basis recognizing that interlesional heterogeneity of response occurs in individual patients. Per-lesion comparisons were performed two-fold: first, independence of individual lesion response from global patient response was assumed and measurements were performed as described for per-patient response analysis. Second, dependence of individual target lesion response on global patient response was assumed. For this scenario, a multi-level model including a random intercept to account for the nested nature of individual lesion measurement analysis was employed.

## Results

### Study population

Three patients were excluded from the parent trial and consequently from this analysis: one for diagnosis of new liver metastases on baseline MRI, one for having received chemotherapy prior to trial inclusion, and one could not tolerate WB-MRI. Eventually, thirty-six patients who received radium-223 and had baseline and follow-up WB-MRI were included for the final analysis. Thirty patients had both baseline 18F-NaF and 18F-choline PET/CT PET. Of the remaining six patients, three patients had only 18F-NaF PET/CT and three patients had only 18F-choline PET/CT PET.

### Per-patient response analysis

Baseline MRI and PET patient parameter measurements are summarized in Table 2. Overall, 14 patients were MRI responders. Ten/14 responders showed the largest gADC increase at the third follow-up MRI (mean gADC increase 72%), 3/14 at second follow-up (mean 56%), and one patient with the first follow-up MRI (36%). Average time to best response among responding patients was 17.8 weeks. Mean “best patient response” gADC increase was 66%. Among all non-responders, the mean “best patient response” gADC increase was 15.5%. Responders and non-responders did not show significant differences in baseline gADC or baseline tDV.

**Table 2:** Baseline per-patient imaging parameters

Parameter^†^	Summary measure	Responder	Non-responder	*p*-value^†^
Global ADC in µm^**2**^**/s**	Mean (SD)	867 (126)	902 (123)	0.422
	Median (IQR)	860 (773–935)	912 (831–988)	
Total disease volume in mL	Mean (SD)	174 (262)	282 (382)	0.343^¥^
	Median (IQR)	73 (31–169)	88 (53–373)	
18F-NaF PET SUVmax	Mean (SD)	61.1 (24.4)	41.2 (21.6)	0.008^¥^
	Median (IQR)	56.0 (48.7–70.8)	38.7 (24.5–47.5)	
18F-choline PET SUVmax	Mean (SD)	10.2 (2.8)	8.6 (3.2)	0.164
	Median (IQR)	9.8 (8.0–12.5)	8.6 (5.7–11.0)	

18F-NaF and 18F-choline PET SUVmax values were derived by calculating the average SUVmax across all five target lesions per patient; from these patient average SUVmax values, the mean and median values across the study population were calculated and are shown in this table

^†^*p*-value from *t*-test unless otherwise indicated

^¥^*p*-value from rank-sum due to non-normality

For both 18F-NaF PET/CT and 18F-choline PET/CT, 13/33 patients (39%) were MRI responders and 20 (61%) non-responders, respectively.

#### 18F-NaF PET/CT

**Fig. 3:**
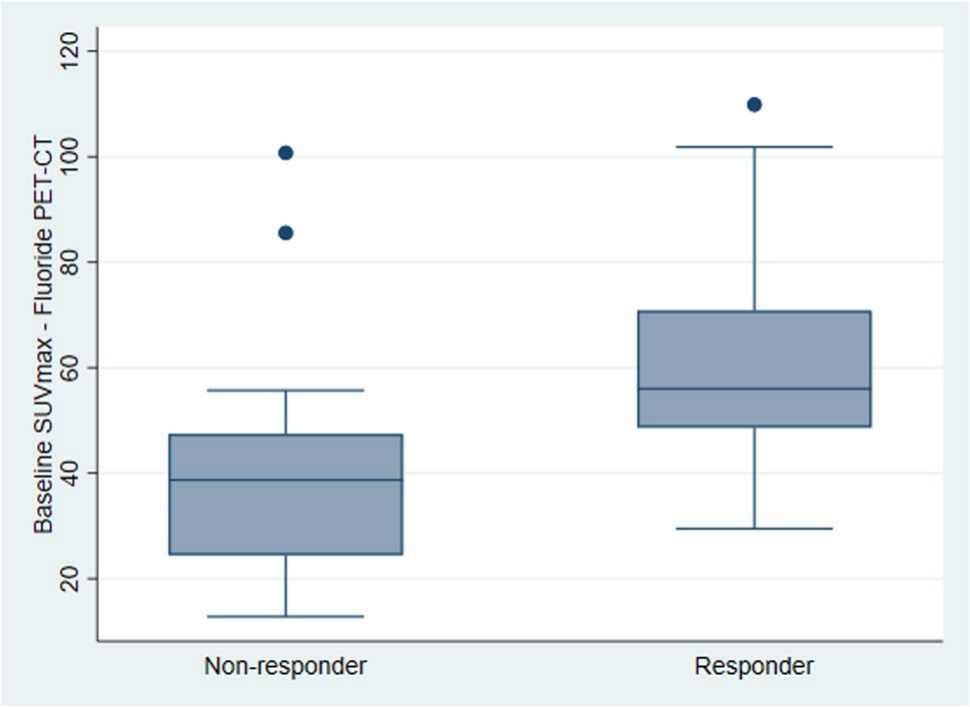
Boxplot visualizing the difference in patient median baseline 18F-NaF PET SUVmax, derived from target lesion measurements, between non-responders (median: 38.7) and responders (median: 56.0)

The median baseline target 18F-NaF PET SUVmax across all available study patients was significantly higher in responders (median: 56.0) compared to that in non-responders (median: 38.7, *p* = 0.008; Fig. 3).

**Fig. 4:**
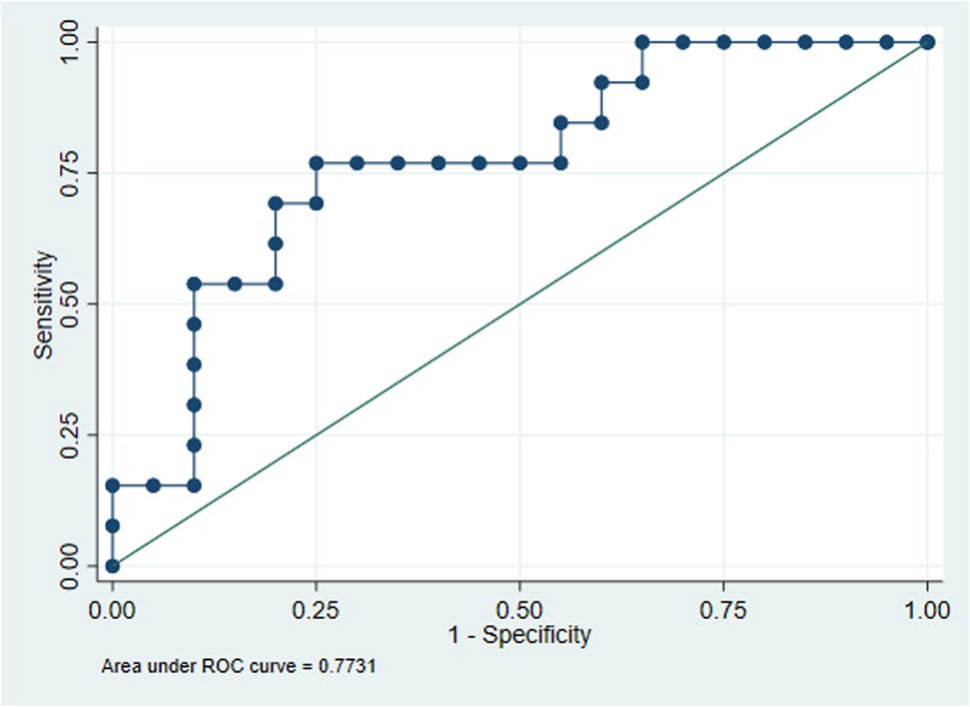
Receiver operating characteristic curve for median baseline 18F-NaF PET SUVmax, derived from target lesion measurements, for discrimination between responders and non-responders

**Fig. 5:**
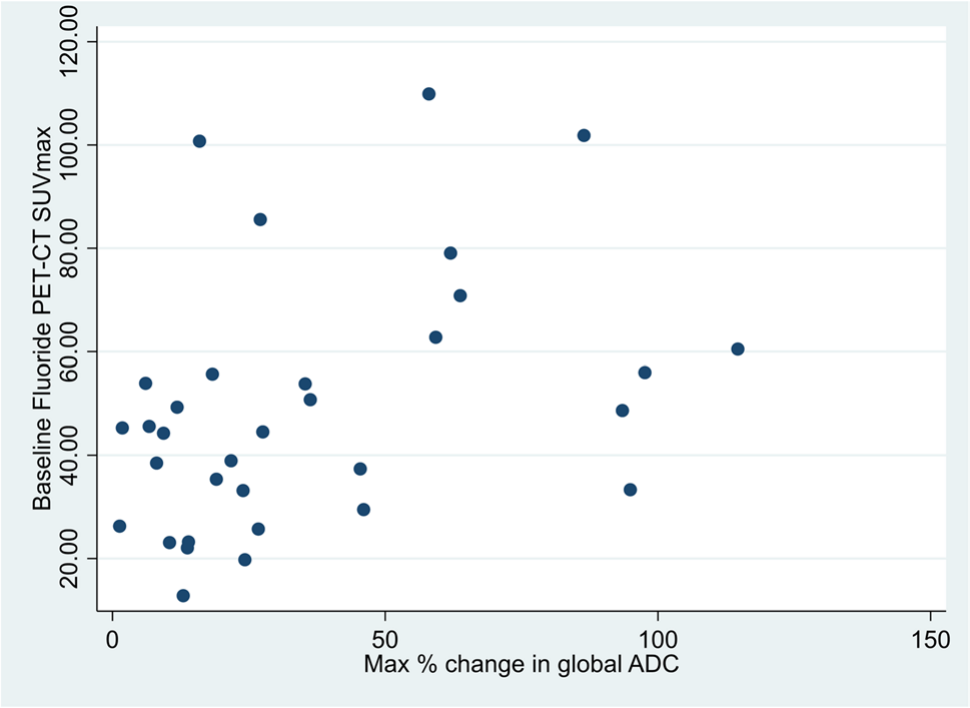
Scatterplot visualizing the correlation between baseline SUVmax, derived from target lesion measurements, and global ADC maximum change between baseline and the follow-up MRI with the largest global ADC increase (best patient response), significant positive correlation (rho = 0.42)

ROC curve analyses revealed an AUC of 0.77 (95% CI 0.61–0.94; Fig. 4). An optimized threshold value of 48.7 SUVmax identified responders with 77% (95% CI 46–95%) sensitivity, 75% (95% CI 51–91%) specificity, 67% (95% CI 38–88%) positive predictive value (PPV), and 83% (95% CI 59–96%) negative predictive value (NPV). Significant positive correlation was found between 18F-NaF PET baseline SUVmax and median gADC change (rho = 0.42, *p* = 0.015), which is presented in Fig. 5.

#### 18F-choline PET/CT

There was no significant difference in baseline target median SUVmax values between responders (mean = 10.2) and non-responders (mean = 8.6, *p* = 0.164).

### Per-lesion analysis

Across all study subjects, 133 target lesions were chosen, resulting in an average of 3.7 lesions per patient. Baseline MRI and PET target lesion parameters are summarized in Table 3.

**Table 3:** Individual target lesion baseline characteristics

Parameter	Summary measure	Responder	Non-responder	*p*-value^†^
ADC in µm^2^/s	Mean (SD)	827 (157)	905 (163)	0.005
	Median (IQR)	822 (725–942)	902 (802–1034)	
18F-NaF PET SUVmax	Mean (SD)	56.1 (32.2)	37.0 (22.8)	0.0001^¥^
	Median (IQR)	51.6 (33.3–70.8)	31.8 (21.3–50.1)	
18F-choline PET SUVmax	Mean (SD)	9.3 (3.8)	9.4 (3.7)	0.922
	Median (IQR)	9.0 (6.5–12.1)	9.5 (6.5–11.6)	

Mean and median SUVmax are calculated across all target lesions

^†^*p*-value from *t*-test unless otherwise indicated

^¥^*p*-value from rank-sum due to non-normality

Seventy-six (59%) targets were defined as responding and 57 (41%) as non-responding on MRI. Among responding target lesions, 37/76 showed the largest ADC increase on the third follow-up MRI (84% ADC increase), 33/76 after the second follow-up (56%), and 6/76 after the first follow-up MRI (47%). Mean “best lesion response” ADC increase was 69%. Among non-responding targets, the largest mean ADC increase was 16%.

One hundred and twenty target lesions were measured on 18F-NaF (68 (57%) MRI responders, 52 (43%) non-responding) and 119 target lesions (69 (58%) MRI responders, 50 (42%) non-responding) on 18F-choline PET.

### Per-lesion analysis assuming independence of individual lesion and global patient response

#### 18F-NaF PET/CT

**Fig. 6:**
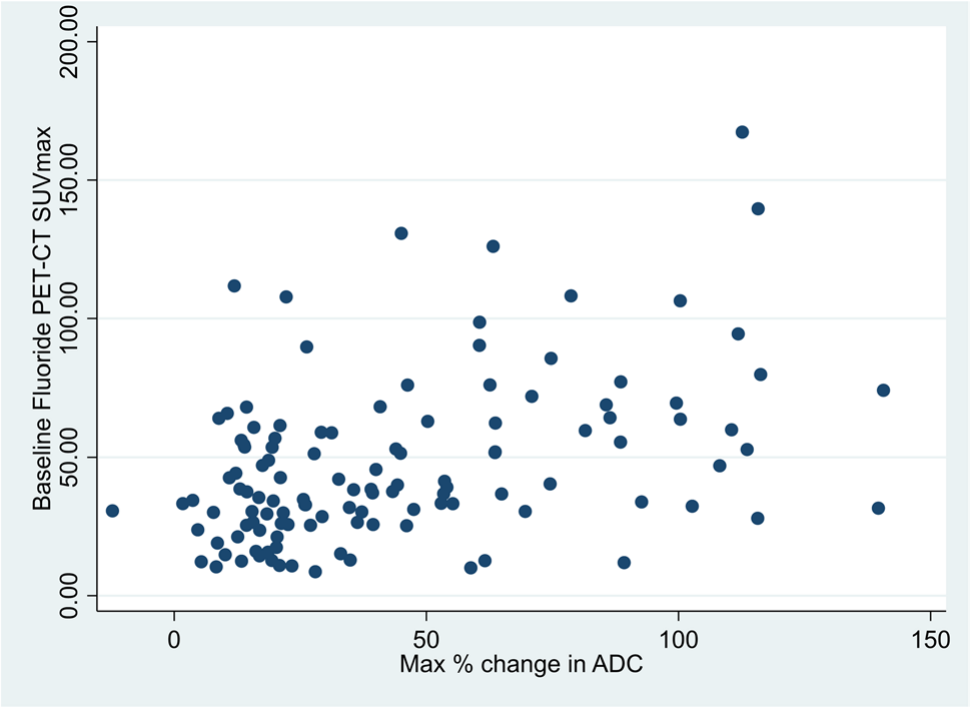
Scatterplot visualizing the correlation between individual target lesion average baseline SUVmax and individual target ADC maximum change between baseline and any follow-up MRI (best target lesion response), significant positive correlation (rho = 0.39)

Baseline 18F-NaF SUVmax was significantly higher in responding (median = 51.6) compared with that in non-responding metastases (median = 31.8, *p* = 0.001). ROC curve analyses revealed an AUC of 0.70 (95% CI 0.61–0.80). An optimized threshold value of 36.8 SUVmax identified responding lesions with 72% (95% CI 59–82%) sensitivity, 63% (95% CI 49–75%) specificity, 69% (95% CI 56–79%) PPV, and 66% (95% CI 52–79%) NPV. Significant positive correlation was found between target lesion 18F-NaF PET baseline SUVmax and median ADC change (rho = 0.39, *p* < 0.001), which is presented in Fig. 6.

#### 18F-choline PET/CT

There was no significant baseline 18F-choline PET SUVmax difference between responding (median = 9.0) and non-responding lesions (median = 9.5, *p* = 0.922).

### Per-lesion analysis assuming dependence of individual lesion on patient response

The mixed effect model revealed no significant fixed effect accounting for baseline 18F-NaF PET SUVmax difference between responding and non-responding lesions (odds ratio = 1.02; 95%CI = 1.00 to 1.05; *p* = 0.051), while the random intercept contributing to value difference was significant (standard deviation of random intercept = 1.5; *p* = 0.002).

No significant fixed effect was shown for 18F-choline PET SUVmax difference between responders and non-responders (odds ratio = 0.98; 95%CI = 0.83 to 1.16; *p* = 0.844), while the random effect was significant (standard deviation of random intercept = 1.84; *p* < 0.001).

## Discussion

We found that baseline 18F-NaF PET SUVmax can distinguish between mCRPC responders and non-responders to radium-223, defined by gADC increase ≥ 30% during treatment either on a per-patient or per-lesion basis. The mean 18F-NaF PET SUVmax across 5 target bone metastases was significantly higher in responders. A SUVmax threshold of 48.7 yielded 67% PPV and 83% NPV to identify responders. A positive correlation was observed between bone lesion baseline 18F-NaF PET SUVmax and interval ADC increase during therapy. This supports the association of greater radium-223 uptake and tumor cell kill in disease with higher baseline tracer uptake measured by SUVmax. No such relationship was observed for SUVmax measurements on the contemporaneous 18F-choline PET/CT examinations.

Our findings support the hypothesis that metastatic disease with higher baseline 18F-NaF PET SUVmax is more likely to respond to radium-223 therapy, while less tracer-avid metastases do not, and that a mCRPC patient with a higher average SUVmax across his bone disease is more likely to benefit from radium-223 therapy. This is in keeping with the findings of a retrospective study of six mCRPC patients. Higher baseline SUVmax values on 18F-NaF PET were measured from bone metastases, which showed response defined as a SUVmax decrease of ≥ 30% after radium-223 injection. A baseline ≥ 53 SUVmax threshold identified responders with 90% sensitivity and 85% specificity [17]. A separate study analyzing repeatability determined a 15% limit of agreement for 18F-NaF PET SUVmax measurements [13]. As such, the 48.7 SUVmax threshold determined in our study is within the limit of agreement and comparable to the 53 SUVmax threshold described previously. 

The lower 77% sensitivity and 75% specificity observed in our study could be related to the use of DWI-derived ADC measurements as the reference standard to assess response, compared with 18F-NaF SUVmax decrease by ≥ 30% in the study by Letellier et al [17]. Nonetheless, our study highlights that SUVmax measurement on 18F-NaF PET-CT is a potentially useful predictive biomarker for response to radium-223 treatment, even when response is defined by another imaging technique. In another study of 29 lesions in five patients, the baseline 18F-NaF SUVmean was positively correlated with radium-223 dosimetry and per-lesion response, thus further supporting the role of baseline 18F-NaF PET/CT as a predictive biomarker to radium-223 therapy [24]. 

Baseline 18F-choline PET SUVmax was not associated with response to treatment. Previous studies in mCRPC patients receiving enzalutamide have identified negative correlation between SUVmax and progression-free and overall survival [21, 22]. In a prospective multicenter study including 40 mCRPC patients receiving radium-223 therapy, a significant negative correlation between baseline SUVmax of the five most metabolically active metastases and overall survival was found. Lesion measurements larger than the determined 5.95 SUVmax and 4.75 SUVmean threshold were associated with worse patient outcome [25]. By contrast, a more recent retrospective study of 20 mCRPC patients receiving radium-223 therapy found that the baseline SUVmax summarized across all lesions had no significant predictive value, which is in keeping with our findings [20]. In their seminal paper on radium-223 therapy in mCRPC, including 921 patients, Parker et al found a 30% reduction of the risk of death when compared with the placebo group. Patients receiving radium-223 had a 47% response rate as defined by total serum alkaline phosphatase reduction < 30% during therapy and a 34% response rate when defined by normalization of serum alkaline phosphate levels [4]. The overall patient response rate of 39% (defined by gADC increase) in our study is comparable to these findings corroborating ADC as a meaningful surrogate for response evaluation in mCRPC patients.

This promotes the general applicability of our findings and further supports utilizing the WB-MRI parameter gADC as a response biomarker in mCRPC metastases on a per-patient basis [26, 27].

Per-lesion analysis was performed two-ways in our study. First, target lesion measurements were analyzed independently from global patient response status. The second method assumed a direct relationship between global patient response and individual lesion response. The first approach confirmed the results found on a global patient level, with significant association of the baseline 18F-NaF PET SUVmax for lesion response defined by ADC. Conversely, the second approach narrowly failed to show a significant effect of response categorization on 18F-NaF SUVmax (*p* = 0.051), attributing significant cause of SUVmax differences to random effects (*p* = 0.002). These random effects may reflect the inter-tumoral heterogeneity of mCRPC bone metastases, which become more pronounced when only five target lesions are chosen for per-lesion analyses [28]. As such, we believe significant predictive value of baseline SUVmax for individual target lesion response to radium-223 can be assumed.

This study has limitations. First, only 36 patients were recruited in this prospective multicenter study. Second, SUVmax was the only recorded PET parameter. Other measurements such as SUVmean may provide additional information. However, SUVmax is one of the most common and well-established imaging biomarkers with good reproducibility, which is easily obtained without advanced software, facilitating its application in clinical practice. Third, we defined response by ADC change on a per-patient and per-lesion basis. However, there is lack of a universally accepted gold standard for response evaluation in bone metastases. Finally, target lesions were chosen on MRI, as response was defined by ADC. Choosing lesions on baseline 18F-NaF PET/CT and using the PET imaging to define response could also alter the results of the study.

In conclusion, 18F-NaF but not 18F-choline PET baseline SUVmax of target mCRPC bone disease showed significant association with response to radium-223 defined by ADC change, which may be further investigated as a predictive biomarker for treatment response.

## Abbreviations

gADC: Global apparent diffusion coefficient
mCRPC: Metastatic castration-resistant prostate cancer
NaF: Sodium fluoride
tDV: Total disease volume
VIBE: Volume-interpolated breath hold examination
WB: Whole-body

## Declarations

### Guarantor

The scientific guarantor of this publication is Professor Dow-Mu Koh.

### Statistics and biometry

One of the authors has significant statistical expertise.

### Study subjects or cohorts overlap

No study subjects or cohorts have been previously reported; the subjects will be reported as part of the trial.

### Methodology

• prospective

• observational

• multicenter study
